# Predictors of under-weight among children younger than 24 months in Nimule Border Town, Eastern Equatoria State, South Sudan: a community-based cross sectional study

**DOI:** 10.1186/s12889-024-18836-9

**Published:** 2024-05-25

**Authors:** Francis Eriga, Godfrey Gulom, John Bosco Alege

**Affiliations:** 1 Institute of Public Health and Management, Clarke International University, Kampala, Uganda; 2 Department of Family Medicine, School of Health Sciences, Community Health and Epidemiology, Kenyatta University, Nairobi, Kenya

**Keywords:** Birth, Weight, Low, Children under 24 months, Determinants

## Abstract

**Background:**

Underweight is a public health problem globally, and more severe in South Sudan with wide sub-regional differences. In Nimule border town, which represents other border towns in South Sudan, data on underweight among children below two years is unavailable. Thus, this study set out to assess predictors of underweight among children younger than 24 months in Nimule border town, Magwi County, Eastern Equatoria State, South Sudan.

**Methods:**

An analytical cross-sectional study was conducted in Nimule boarder town targeting 390 children younger than two years. Systematic sampling and simple random sampling methods were used. Data were collected through a researcher-administered questionnaire with both open and closed ended questions. Data was entered in EpiData and then exported into Statistical Package for Social Sciences research (SPSS) version 20 for analysis. Descriptive data analysis was conducted and data were summarized into frequencies and percentages, means with standard deviations, and medians with interquartile ranges. Bivariate analysis was conducted with the Chi-squared and Fisher's exact tests for categorical independent variables, and the student’s t-test for numerical variables. Finally, multivariate analysis was done via logistic regression analysis and results were stated as odds ratios (OR) with corresponding 95% confidence interval (CI). The level of statistical significance was set at 5%.

**Results:**

Out of the total 390 participants, 112 (28.7%) were under weight. The study showed primary (aOR, 0.38; 95% CI, 0.12–1.18; *P* = 0.095) and secondary (aOR, 0.37; 95% CI, 0.12–1.17; *P* = 0.091 levels of education were associated with underweight but not tertiary level of education (aOR, 0.76; 95% CI, 0.21–2.74; *p* = 0.671). Household income of 5000 to 10,000 (aOR, 0.26; 95% CI, 0.10–0.68; *P* = 0.006) and above 10,000 (aOR, 0.11; 95% CI, 0.04–0.28; *P* < 0.001) South Sudanese pounds, supplementary feeding before 6 months (aOR; 0.01; 95% CI, 0.02–0.05; *P* < 0.001) were associated with underweight and irregular hand washing (aOR; 2.17; 95% CI, 1.14–4.11; *P* = 0.018) was associated with increased odds of underweight.

**Conclusions:**

This study established a high prevalence of underweight. Maternal level of education particularly primary, secondary, higher household incomes in excess of 5,000 South Sudanese pounds reduced the risk of underweight. While irregular hand washing was a risk factor for underweight.

## Background

According to the Global Nutrition report (2018), malnutrition remains a severe problem, especially under nutrition, which is one of the major causes of child mortality globally. Besides, it seems that the world is not on track to achieve the target of reducing malnutrition by 40%, as a result, it remains unacceptably high across all regions of the world [1]. According to the Global burden of diseases, approximately 200 deaths per 100,000 in children less than five years are due to childhood under nutrition, equivalent of 21% total deaths in this age bracket [2]. In South East Asia, under nutrition continues to be an alarming public health concern especially in Pakistan, Bangladesh, and India [3]. In Pakistan, under nutrition is responsible for 428 deaths per 100,000 in children less than five years [2]. Besides, A Save the Children report (2012) in the UK revealed that 96% of the children less than two years do not receive the adequate diet recommended [4]. Similarly, Indonesia has been ranked the fifth highest of stunted children globally, as a result, one in every three children are stunted implying 9.7 million children under five are malnourished [5]. In the same report, about three million under-five children are wasted and two in every three children below two years of age are anemic. There is no clear data for monitoring progress on the target of reducing the incidence of low birth weight (LBW) by 30%. In the WHO African Region, 26 of the member countries with data have LBW rates in excess of 10%. This is because population groups with high socioeconomic status are more likely to deliver in maternity facilities under the care of skilled birth attendants where birth weight is measured, compared to groups with lower socioeconomic status who often deliver at home and are more likely to have babies with low birth weight [6]. A population-based subnational study conducted in Pakistan established that a child whose birth order was three or more is two times more likely to be underweight compared to a child of a lower birth order. Other factors being diarrhoea that significantly increased the likelihood of the child being underweight, child size, for instance, a child with a size smaller than average at the time of birth was more than two times likely to be moderately underweight compared to a child with an average or larger than average size at the time of birth [28]. Among most Sub-Saharan African countries, the prevalence of wasting among children below five years of age remained below the emergency threshold level, though at the poor nutritional threshold level of 6.5% for East Africa [7]. A study conducted in Kenya by Helen Guyatt et al*.,* (2020) on prevalence and predictors of underweight among children under 2 years established that 23% of the 1004 children with anthropometric data were stunted, 10% were underweight and 6% experienced wasting. Children who were 24 months and older were found to be stunted and underweight. Residing in a poor household with more than one child under 2 years of age were also significant risk factors for being underweight. Although 43% of children did not receive the minimal acceptable diet, this was not a significant factor associated with undernutrition. When age was removed as a covariate in children aged 12–23 months, being male resulted in a significantly higher risk of being stunted [8].

Similarly, a study conducted in Ethiopia also found that age (months) of child, illness, growth monitoring, immunization, and control of infectious diseases were the child specific predictors of under-weight. While maternal decision-making power, was a strong influencer underweight among children, alongside maternal education, employment/occupation, and household income as the other independent contributors of under-weight [29]. In addition, Malnutrition in developing countries is accountable for about one in five of all disability-adjusted life years, and Nigeria was ranked amongst the ten countries with the highest prevalence of underweight, stunting and wasting in children below five years [9].

In Uganda, the burden of childhood malnutrition is more prevalent in rural areas compared to the urban areas, 29% (2.2 million) of the children suffered from stunting [low height-for-age], Uganda Bureau of Statistics [10]. While in terms of economic loss yearly, Uganda loses 1.8 trillion shillings equivalent of 6% of the country's Gross Domestic Product (DGP) in treatment, management, and prevention of childhood malnutrition [11]. In addition, childhood malnutrition is associated with premature deaths and consequential life-course perspective in economic development [12].

In South Sudan, malnutrition is a serious public health problem where approximately 200,000 children below five years are at risk of malnutrition [5]. As a result, South Sudan ranks 15th highest in the world in terms of mortality rates for children below five years [13]. Similarly, one in every seventh South Sudanese child dies before their fifth birthday due to diarrheal diseases and the burden of malnutrition is substantial with its rate-exceeding the 15% threshold of WHO [14]. Besides, in 2014, the burden of moderate acute malnutrition increased by 44.7% from 123, 3832 to 675, 4003, and over 900,000 children were severely malnourished; hence, the burden is more in Jonglei, Upper Nile and Unity states [13]. In Torit State particularly Nimule boarder town, there is limited information on the burden of malnutrition especially underweight among children below two years of age.

## Methods

### Study setting and design

This study used analytical cross-sectional study design to determine the proportion of children below 24 months who are underweight in Nimule border town. The study also determined factors associated with underweight. The exposure and the outcome data was collected at the same point in time. This study was conducted in Nimule border town, Magwi County, Eastern Equatoria State, South Sudan. Nimule border town is located in Magwi county, Eastern Equatoria State, South Suan bordering the Republic of Uganda. There is one county civil hospital run by the government, one Primary Healthcare Centre under the Catholic Diocese of Torit and several private clinics. The town is relatively peaceful, however, there is not sufficient food supply to meet the ever-growing population that is cosmopolitan [15].

### Sample size, and sampling procedure

This community-based study targeted mothers and caretakers of children younger than 24 months in Nimule border town, Magwi County, Eastern Equatoria, South Sudan. Mothers within the reproductive age (15–49 years) and caretakers of children less than two years who had growth monitoring cards and voluntarily consented to participate in the study, and had spent at least three months in the study area were eligible to participate in the study.

Open Epi software was used for the sample size estimation. The sample size of 390 was established on expected result estimation of about 50% positive (underweight) and 50% negative (normal weight) with 95% confidence that the true frequency lies between 45 and 55% since there is no previous estimate of underweight among children younger than 24 months in Nimule border town. Probability sampling technique was used in order to provide equal opportunity for the participants to participate in the study. Nimule Border town has seven *Bomas* out of which four were randomly selected for the study. To obtain study participants for data collection, a sampling frame was generated from the estimated number of households in the four *Bomas,* and seventeen villages. Then systematic random sampling technique was used to obtain the interval. Thereafter, a table of random numbers were generated against the number of the household through simple random techniques to identify the number where the interval will start. For households that had more than one eligible child, a lottery method was considered to select one child for the study.

In this study, data were obtained from both primary and secondary sources. This involved face to face interviews with the participants and child’s information were obtained from the growth monitoring cards.

### Data collection and quality control measures

A researcher administered interview was used to collect data. In this method, the researcher conducted face to face interviews with the respondents so that those who were unable to read and write were easily helped. Besides, this method also reduced the chances of high non-response rate by the target population. Semi Structured questionnaires were used to obtain reliable data from study participants. This tool enabled the researcher to generate well-structured set of responses that were easily coded and statistically analyzed as well as generating in-depth knowledge, attitude and opinions from the respondents to compliment and strengthen the result that were generated from the study. This kind of questionnaire were easily answered by the study participants, and the different responses were easily compared. The research team ensured that data were checked daily for consistency and completeness. Questionnaires with missing data were discarded.

### Measurements

Underweight was categorized using the WHO growth chart for children and is defined as being <  − 2 SD from the median reference weight for age [16]. Weight were measured using electronic weighing scales (kg). Heights and lengths measurements were carried out using measurement boards (stadiometers in cm) while laying down given the fact that the children are less than 24 months or less than 85 cm, Presence of pitting oedema was checked by applying a gentle pressure with the thumb for 3–5 s. It was registered as “0” if no pitting was detected on the feet [17]. The child’s determinants like age was measured in months, child sex as male and female, Birth Interval was measured in months before the next child, immunization status verified from vaccination cards to establish whether immunized or not and child morbidity was measured by the periods or frequency of illness that the child experienced based on a recall by the mother or care giver. While maternal factors like age was calculated in years, marital status was either single parent or married, Weight of the mother was determined by the body mass index, place of residence was either urban or peri-urban, education level was determined by asking whether the mother or caretaker completed primary, secondary, tertiary/university or none, and household income low or high was determine by the World Bank cut off point of 2 dollars a day per person.

The nutritional practices like dietary practices was measured by the WHO defined minimum dietary diversity as the proportion of children aged (6–23 months) who received foods from at least four out of seven food groups. The seven food groups used in defining children’s minimum dietary diversity indicator are: (i) grains, roots and tubers; (ii) legumes and nuts; (iii) dairy products; (iv) flesh foods (meat/fish/poultry) (v) eggs (vi) vitamin A rich fruits and vegetables; and (vii) other fruits and vegetables. Breastfeeding practices was on demand and the months of exclusive breastfeeding, age of complementary feeding was either before or after six months, age at weaning is calculated by months at weaning, and Hygiene practices was determined by asking about the sanitation facilities like use of latrine and hand washing facilities after latrine use.

### Data analysis

Data were doubled checked for completeness and coded before entry into EPI data and SSPS version 20 for analysis. ENA SMART was used to determine the prevalence of underweight. Descriptive data analysis was performed and data were summarized into frequencies and percentages, means with standard deviations, and medians with interquartile ranges. Bivariate analysis was performed with the Chi-squared and Fisher's exact test for categorical independent variables, and the student’s t-test for numerical variables. While multivariate analysis was done via logistic regression analysis and results were stated as odds ratios (OR) with corresponding 95% confidence interval (CI). The level of statistical significance was set at 5%.

The researcher conducted one day training for two research Assistants on how to conduct interviews, carry out measurements and also briefed them on research ethics, especially on how to seek for consent from study participants, and maintain confidentiality throughout the data collection process. The standardized translated questionnaires from English to Madi and local Arabic were then used for by the research assistants for data collection. Completed questionnaires were checked daily for accuracy, consistency and completeness. Pre-testing of at least 10% of the sample size questionnaires was conducted in the Dioceses of Torit primary Health Care center (PHCC) in Nimule boarder town prior to data collection.

## Results

### Prevalence of underweight based on weight-for-age z-scores by sex

**Fig. 1:**
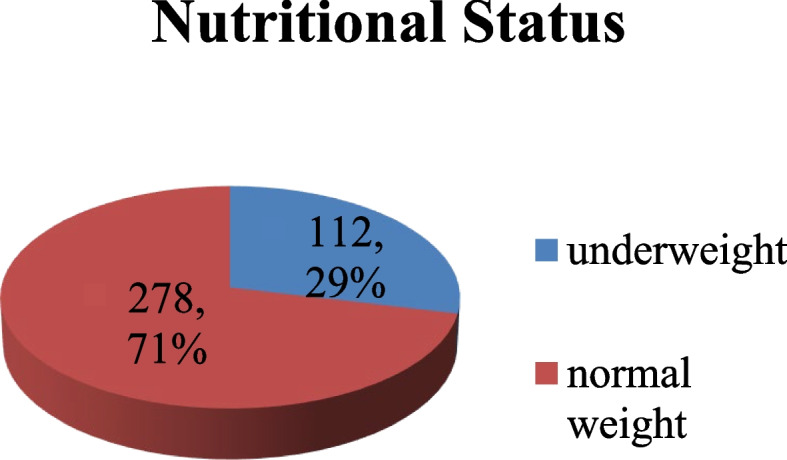
According to the result presented in (Figure 1), 112(29%) of the children were underweight while 278(71%) had normal body weight children. Among the underweight children, 32.6% were boys as compared to girls 24.9%. In addition, the study found that 21.8% of the boys had moderate underweight unlike 10.9% who were severely underweight. Regarding girls, 18.3% were moderately underweight as compared to the 6.6% who were severely underweight (for further details see Table 1 below).

According to the result presented in (Fig. 1) below, 112 (29%) of the children were underweight while 278(71%) had normal body weight.

### Prevalence of underweight by age, based on weight-for-age z-scores

Among the underweight children, 32.6% were boys as compared to girls 24.9%. In addition, the study found that 21.8% of the boys had moderate underweight unlike 10.9% who were severely underweight. Regarding girls, 18.3% were moderately underweight as compared to the 6.6% who were severely underweight (see Table 1 below).

**Table 1:** Prevalence of underweight based on weight-for-age (months) z-scores by sex

Variable	All *n* = 390	Boys *n* = 193	Girls *n* = 197
Prevalence of underweight (< -2 z-score)	112(28.7% (24.5 -33.4 95% C.I.)	62 (32.6% (26.4—39.5 95% C.I.)	(50) 24.9%(19.4 -31.4 95% C.I
Prevalence of moderate underweight (< -2 z-score and > = -3 z-score)	78 (20.0% (16.3 -24.3 95% C.I.)	42 (21.8% (16.5—8.1 95% C.I.)	36 (18.3% (13.5—24.3 95% C.I.)
Prevalence of severe underweight (< -3 z-score)	34 (8.7% (6.3—11.9 95% C.I.)	20 (10.9%(7.2—16.1 95% C.I.)	(14) 6.6%(3.9—11.0 95% C.I.)

The prevalence of underweight by months indicates that 15(5.6%) of the children 6 to 12 (months) were severely underweight as compared to the 20(16.1%) aged 13 to 24 months. while, 46(17.3%) of the children aged 6 to 12 months were moderately underweight unlike 30(24.2%) aged 13 to 24 months (refer to Table 2 above).

**Table 2:** Prevalence of underweight by age (months), based on weight-for-age z-scores

Variable	Total	Severe underweight (< -3 z-score)	Moderate underweight (> = -3 and < -2 z-score)	Normal (> = -2 z score)	Oedema
Age	No	No.(%)	No.(%)	No.(%)	No. (%)
6–12	266	15(5.6)	46(17.3)	205(77.1)	0(0.0)
13–24	124	20(16.1)	30(24.2)	74(59.7)	0(0.0)
**Total**	**390**	**35(9.0)**	**76(19.5)**	**279(71.7)**	**0(0.0)**

### Child’s determinants associated with underweight

The frequency distribution of the child determinants presented in (Table 3 above) indicated that 193 (49.5%) of the respondents had baby boys while 197(50.5%) had baby girls. Regarding birth interval, 150(38.5%) of the respondents had spaced their children less than 12 months while 48(12.3%) were spaced 25 months and above. In addition, almost all 376(96.4%) of the children had suffered from any disease in the past six months. 204 (52.3%) of the children had suffered from diarrhea, 31(7.9%) acute respiratory infection, 35(9.0%) upper respiratory infection, 256(65.6%) malaria and 52(13.3%) suffered from other diseases. Besides, 281(72.1%) of the had received recommended immunization services as compared to the 109 (27.9%). The study established that all the children had received polio and BCG vaccines. Meanwhile, 387(99.2%) of the children were given hepatitis B vaccine, 282(72.3%) measles, 385(98.7%) DPT and 382(97.9%) had whooping cough.

**Table 3:** Child’s determinants associated with underweight

Variables	Underweight No. (%)	Normal weight No. (%)	Total No. (%)	*P*-value
Sex of the Child				0.141
Male	62(55.4)	131(47.1)	193(49.5)	
Female	50(44.6)	147(52.9)	197(50.5)	
Age of the child				0.738
6 to 12 months	75(67.0)	191(68.7)	266(68.2)	
13 to 24 months	37(33.0)	87(31.3)	124(31.8)	
Birth interval				0.196
First birth	41(36.6)	88(31.7)	129(33.1)	
Less than 12 months	42(37.5)	108(38.8)	150(38.5)	
12 months 24 months	21(18.8)	42(63.0)	63(16.2)	
25 months and above	8(7.1)	40(14.4)	48(12.3)	
Child ever suffered disease past six months				0.990
Yes	108(96.4)	268(96.4)	376(96.4)	
No	4(3.6)	10(3.6)	14(3.6)	
Child received recommended Immunization				0.566
Yes	83(74.1)	198(71.2)	281(72.1)	
No	29(25.9)	80(28.8)	109(27.9)	

Source primary field data 2019

The child’s determinants were analyzed to assess their association with underweight in Nimule border town, South Sudan. However, the study finding indicated that none of the child’s determinants had any significant effect on underweight (refer to Table 3 above).

### Maternal factors associated with underweight

Table 4 above presents maternal factors associated with underweight. Among the 390 respondents who participated in this study, 215 (55.1%) were aged between 15 to 24 years while 175 (44.9%) lie between 25 to 59 years with the mean of 1.45 ± 0.498. 359 (92.1%) of the participants were married, while 31(7.9%) were either divorced or separated. 240(61.5%) of the participants lived in peri-urban setting as compared to the 150(38.5) living in urban setting. The majority of participants have basic level of education with only 64 (17.4%) reached tertiary level, 141(36.2%) completed secondary level while 128(32.8%) ended in primary and the remaining 53(13.6%) had no formal education. The findings also indicated that only 112 (28.7%) of the entire participants had household income above 10,000 South Sudan Pounds. The analysis of maternal factors indicated that mother/caretaker’s education level had a significant association with underweight (*P* = 0.011). Monthly household income has also shown a highly significant (*P* =  < 0.001) effect on the prevalence of underweight.

**Table 4:** Maternal factors associated with under weight

Variables	Underweight	Normal weight	Total	*P*-value
	**No. (%)**	**No. (%)**	**No. (%)**	
**Age of the mothers/caretakers**				0.129
** 15 to 24**	55(49.1)	160(57.6)	215(55.1)	
**25 and above**	57(50.9)	118(42.4)	175(44.9)	
**Marital Status**				0.200
** Married**	100(89.3)	259(93.2)	359(92.1)	
** Divorced/separated**	12(10.7)	19(6.8)	31(7.9)	
**Residence**				0.104
** Rural**	76(67.9)	164(59.0)	240(61.5)	
** Urban**	36(32.1)	114(41.0)	150(38.5)	
**Highest level of education**				**0.011**
** Primary**	40(35.7)	88(31.7)	128(32.8)	
** Secondary**	50(44.6)	91(32.7)	141(36.2)	
** Tertiary**	15(13.4)	53(19.1)	68(17.4)	
**Non-formal Education**	7(6.3)	46(16.5)	53(13.6)	
**Monthly household income**				** < 0.001**
** Less or equals 4900**	23(20.5)	131(47.1)	154(39.5)	
** 5000 to 10,000**	31(27.7)	93(33.5)	124(31.8)	
** Above 10,000**	58(51.8)	54(19.4)	112(28.7)	
**Mother’s BMI**				0.613
** Underweight**	10(8.9)	20(7.2)	30(7.7)	
** Normal**	75(67)	200(71.9)	275(70.5)	
** Overweight/obese**	27(24.1)	58(20.9)	85(21.8)	

Source primary field data 2019

However, maternal/caretaker’s factors like age, marital status, residences, and BMI were not associated with underweight.

### Nutritional practices associated with underweight

Table 5 above presents data on nutritional practices associated with underweight in this study. The study established that the majority of the respondents 371(95.1%) fed their children on entirely family food while 19(4.9%) did not. However, 44(11.3%) of the children were fed more than three times a day while less than half 156(40.0%) of them were fed twice a day. Regarding breastfeeding practices, 21(5.4%) of the children were exclusively breastfed while 349(89.5%) had both breast milk and milk substitutes. Besides, more than half 256(65.6%) of the respondents were encouraged by staff to look for signs of breastfeeding. Yet, 171(43.8%) of the respondents were advised every time to see whether the baby seemed hungry as compared to 12(3.1%) were advised to breastfeed two to three times a day. 317(81.3%) of the respondents agreed that they had given anything other than breast milk to their baby. The study also revealed that 35(9.0%) of the mothers practiced exclusive feeding only despite their children being six months and above, 64(16.4%) of the children were started on food supplements before six months, 258(66.2%) started their children on complementary feeding as recommended by WHO at 6 months while 33(8.5%) where started after six months.

**Table 5:** Nutritional practices associated with underweight

**Variables**	**Underweight No. (%)**	**Normal weight No. (%)**	**Total No. (%)**	*P*-Value
Feeding the baby entirely on Family food?				0.812
Yes	107(95.5)	264(95)	371(95.1)	
No	5(4.5)	14(5.0)	19(4.9)	
Method of feeding the baby				0.584
Breastfeeding exclusively	7(6.3)	14(5.0)	21(5.4)	
Mixed method feeding	101(90.2)	248(89.2)	349(89.5)	
Replacement feeding	3(2.7)	7(2.5)	10(2.6)	
No Milk substitutes	1(0.9)	9(3.2)	10(2.6)	
Received health education on breastfeeding				0.196
Yes	79(70.5)	177(63.7)	256(65.6)	
No	33(29.5)	101(36.3)	134(34.4)	
Frequency of hand washing before breastfeeding				** < 0.001**
Always	78(69.6)	100(36.0)	178(45.6)	
Irregularly	34(30.4)	148(53.2)	182(46.7)	
Do not wash	0(0.0)	30(10.8)	30(7.7)	
Breast Feeding duration				0.502
BF < 24 months	64(56.2)	158(56.8)	221(56.7)	
BF 24 months +	49(43.8)	120(43.2)	169(43.3)	
24 months and above	16(14.3)	18(6.5)	34(8.7)	
Safe versus unsafe water sources				0.575
Safe water	110(98.2)	275(98.9)	385(98.7)	
unsafe water	2(1.8)	3(1.1)	5(1.3)	
Age of supplementing child's food(months)				** < 0.001**
Exclusive breastfeeding only	4(3.6%)	31(11.2%)	35(9.0)	
Before 6 months	56(50.0%)	8(2.9%)	64(16.4%)	
At 6 months	52(46.4%)	206(74.1%)	258(66.2)	
At 6 months	0(0.0%)	33(111.9%)	33(8.5%)	

Source primary field data 2019

While according to the bivariate analysis, nutrition practices associated with underweight, frequency of hand washing before breastfeeding and age of supplementing has shown a highly significant (*P* =  < 0.001) association on underweight.

In this study, out of the 9 participants who admitted to have not initiated complementary feeding at 6 months as recommended by WHO, 4 of them acknowledged “*that in South Sudan the price of food items is very high, while the rest attributed this to lack of enough money to purchase food items”.*

Conversely, nutritional practices such as feeding the baby entirely on family food, method of feeding the baby, health education conducted on breastfeeding, breast feeding duration and source of drinking water were not associated with underweight.

### Multivariate analysis of significant factors associated with underweight

Table 6 above presents the model summary of factors associated with underweight in children under 24 months. All variables that had a p-value of less than (0.05) at bi-variate level were fitted in to Binary logistic regression to ascertain the strength association with underweight among children less than 24 months. Mothers/caretakers with primary (aOR, 0.38; 95% CI, 0.12–1.18; *P* = 0.095) and secondary (aOR, 0.37; 95% CI, 0.12–1.17; *P* = 0.091) levels of education had reduced odds of underweight compared to those with no formal education. However, tertiary level of education was not associated with reduced odds of underweight (aOR, 0.76; 95% CI, 0.21–2.74; *p* = 0.671).

**Table 6:** Model summary of factors associated with underweight in children 24 months

Variables	Binary logistic regression analysis					
	**Unadjusted**			**Adjusted**		
	**uOR**	**95% CI**	***P*-value**	**aOR**	**95% CI**	***P*-value**
**Respondents highest level of education**						
Non-formal education	**Ref**			**Ref**		
Primary	0.33	0.14–0.81	0.004	0.38	0.12–1.18	0.095
Secondary	0.27	0.12–0.66	0.215	0.37	0.12–1.17	0.091
Tertiary	0.54	0.20–1-43		0.76	0.21–2.74	0.671
**Respondents monthly household income**						
Min to 4900	**Ref**			Ref		
5000 to 10,000	0.41	0.21–0.80	0.009	0.26	0.10–0.68	0.006
Above 10,000	0.13	0.06–0.25	< 0.001	0.11	0.04–0.28	< 0.001
**Frequency of hand washing before breastfeeding**						
Always	**Ref**			**Ref**		
Irregularly	3.39	2.11–5.46	< 0.001	2.17	1.14–4.11	0.018
Do not wash	1			1		
**Age of supplementing your child’s food**						
Exclusive breastfeeding only	**Ref**			**Ref**		
Less than 6 months	0.02	0.01–0.07	< 0.001	0.01	0.02–0.05	< 0.001
6 Months	0.51	0. 17–1.51	0.225	0.48	0.15–1.54	0.216
Above 6 months	1			1		

In regards to monthly household income, participants with household incomes that ranged from 5000 to 10,000 (aOR, 0.26; 95% CI, 0.10–0.68; *P* = 0.006) and those with incomes above 10,000 (aOR, 0.11; 95% CI, 0.04–0.28; *P* < 0.001) South Sudanese pounds compared with those with incomes less than 5000 South Sudanese pounds had reduced odds of underweight.

Participants who irregularly wash their hands before breast-feeding their children below 2 years had increase odds of underweight compared to those who always wash their hands. However, there was no different between participants who always washed hands before breastfeeding their children and those who do not wash their hands. Participants who began supplementary feeding before six months of age (aOR; 0.01; 95% CI, 0.02–0.05; *P* < 0.001), and those who started at 6 months (aOR, 0.48; 95% CI, 0.15–1.54; *P* = 0.216), had reduce likelihood of underweight compared to those who exclusively breastfed their children. Conversely, there was no association between participants who started supplementary feeding after six months and those who exclusively breastfed their children.

## Discussion

In this study the prevalence of underweight was 28.7% with more boys presenting with underweight compared to girls. A community based cross-sectional study conducted in Western Ethiopia established a much lower prevalence of underweight among under-two children of 8.9%. The study further indicated that the prevalence of underweight among children under the age of one year was 15.1%. Males (9.7%) and were more malnourished than females (8.2%), which is similar to findings from this study. As compared with children in the age group less than six months, the risk of underweight was about 2.6 times higher for children in age groups over one year [18]. On the contrary, a cross-sectional study conducted in South India among 500 participants observed that females were actually found to be more underweight (27.95%) compared to the males 20.29% [19]. These contrasting findings between this study and that in India could be due to some cultural inclination of the communities where these studies were conducted. The cultural preference of a particular gender (girls or boys) on the basis of being sources of wealth or heir to succeed the father means, little attention maybe given to any gender that is not of preference especially in regards to duration of breastfeeding. The need to conceive a baby girl or boy might compel the mother into early cessation of breastfeeding which in turn exposes the child to underweight. Therefore, this study finding is not a surprise because in South Sudan, the girl child is valued more compared to the boy child. These cultures and traditional norms or customs has made the girl child all the time the most vulnerable in society [27].

This study also revealed that underweight was higher in children less than one year compared to those above one year. This is in agreement with findings from a cross-sectional study conducted among 500 participants in rural area of south India which indicated that the prevalence of underweight was higher in children < 1 year of age (37.2%) compared with other age groups [3]. This could be because children above one year have other sources food to feed on apart from the breast milk compared to less than one-year children who mostly depends on breast milk.

This study showed that increase in mothers/caretaker’s level of education was protective against underweight. These findings are consistent with findings from two other similar studies; a cross sectional study, and a case–control conducted in Ethiopia, were in both studies; age of the child and education status of the mother were statistically significant predictors of underweight [18–20].

Similarly, a community based cross-sectional study conducted in Ethiopia among 384 participants also showed that for any unit increase in maternal education (diploma and higher), the odds of their children getting underweight reduces by 81% more than those whose level of education is below diploma level [21]. This could be due to their positive health seeking behaviour because participants who attained at least some formal level of education have a higher chance of being well informed about the ways to provide care to their children, show positive practices with regard to hygiene, breast feeding and empowered decision making in health compared to those who never had any formal education.

Additionally, this study established that households with higher income of at least 5,000 South Sudanese Pounds was associated with reduced likelihood of underweight. A study conducted in Pakistan, revealed a significant association between household wealth and underweight, thus, more cases of underweight were reported in the poorest households compared to those in the wealthiest household [22]. Similarly, findings from this study are consistent with several studies conducted in various countries in Sub-Saharan Africa. For instance, in Malawi, results from analysis of individual-level and community-level effects on childhood undernutrition established that children in communities with a low and medium income had higher chances of being undernourished compared to those from households with low-income levels [23].

The consistency in these findings could be because mothers who have reasonable amount of money are able to afford nutritious foods and provide a balance diet for their children compared to those who do not have any sources of income. Probably that explains why various studies have similar findings on the variable of levels of income or household wealth. This is also in line with opinions of 4/9 of the mothers who had not given any supplementary foods to their children even at 6 months. When asked to explain the reason for not supplementing, they testified *“in South Sudan everything including food items are bought from the market expensively which we cannot afford. A 21-year-old mother said my baby does not want to eat our family food. I wish to make something different like porridge and eggs for her but my husband and I cannot afford enough food for the family to eat even twice a day”.*

This study had proven that mothers or care takers who irregularly wash hands before breastfeeding their children had increased odds of underweight. This finding is similar to those of another study conducted in Ethiopia among 480 participants where it established that Children of caregivers/mothers who did not practice hand washing after latrine use were found to be 6.7 times as likely to be underweight as their counterparts whose caregivers did wash their hands [24]. This could be because one of the major causes of diarrheal disease common in children 6 to 24 months old, happened as a result of bacterial contamination of complementary foods. Mothers or caretakers who irregularly washed before feeding their children are likely to contaminate the foods while preparing it or transmit infection through unclean utensils, such as cups, spoons and bowls, used for storing and serving young children's food.

This study showed that introduction of supplementary feeding before six months of age and at six months was protective against underweight compared to those who exclusively breastfed at six months and those who were introduced complementary feedings after six months. This finding is consistent with the WHO recommendation which states that children should be introduced to supplementary feeding at six months. However, the practice of responsive feeding in WHO complementary guidelines means a child who expresses interest for food even before six months can receive supplementary feeding. This is because at 6 months, the child’s requirements for energy and nutrients starts to exceed what is provided by breast milk [25]. On the contrary, a cross sectional study conducted across Lebanon among 1000 participants, indicated no risk of diarrhoeal diseases associated with the early introduction of nonmilk supplements [26].

In conclusion, this study showed that the prevalence of underweight in children less than 24 months in Nimule town council is high (28.7%). This result falls within WHO underweight high prevalence cut-off values (20–29%) for public health significance. As the mothers or caretaker’s level of education reduces the chances of their children becoming underweight. Monthly household income of at least 5,000 South Sudanese pounds was associated with reduced likelihood of underweight in children. The children of mothers/care takers who irregularly washed hands before breastfeeding their children had increased odds of underweight and the introduction of supplementary feeding before six months of age and at six months was protective against children being underweight.

Findings from this study strengthens the evidence base for MoH Government of South Sudan and the development/Implementing partners to focus on nutrition in children below two years of age. There is need to strengthen the affirmative action efforts to empower the girls and women, through education for instance. Empowerment of girls through formal education is beneficial for maternal and child health outcomes immediately or later in their life. Importantly, empowerment of girls through formal education also increases their chances for jobs and hence improves their earning which is preventive against underweight. Alternatively, this study also recommends for multi sectorial approaches targeting women’s socioeconomic status by introducing income generating activities that could improve household food security. There is need to have access to safe and clean water and to educate households and community members on personal hygiene so as to cut down the fecal oral transmission of diseases among children. Finally, children should be introduced to supplementary feeding at six months as recommended by WHO. However, in line with IMCI guidelines also, when a child expresses interest in food, he/she can be introduced into supplementary feeding before six months since both are protective against underweight.

## Data Availability

The dataset for this study is available and shall be provided in a separate file upload.

## Abbreviations

W H O: World Health Organization
UNICEF: United Nation International Children’s Emergency Funds
WFP: World Food Program
UBOS: Uganda Bureau of Statistics
MOH: Ministry of Health
SMOH: State Ministry of Health
GBD: Global burden of diseases
UK: United Kingdom
BMI: Body Mass Index
SSP: South Sudanese Pounds
SCI: Save the children International
SPSS: Statistical Package for Social Scientist
CDOT: Catholic Dioceses of Torit
CHD: County Health Department
NH: Nimule Hospital
PHCC: Primary Health Care Centre
PHCU: Primary Health Care Units
